# The role of IL-33/ST2 signaling in female reproductive diseases

**DOI:** 10.3389/fcell.2026.1808099

**Published:** 2026-06-12

**Authors:** Ningzhen Zhang, Maoxing Tang, Mingwei Chen, Yuhua Shi

**Affiliations:** 1 Department of Obstetrics and Gynecology, Center for Reproductive Medicine, Nanfang Hospital, Southern Medical University, Guangzhou, China; 2 Department of Reproductive Medicine, Guangdong Women and Children Hospital, Guangzhou, China

**Keywords:** endometriosis, fibrosis, IL-33/ST2, immune tolerance, macrophage, polycystic ovary syndrome, primary ovarian insufficiency, recurrent miscarriage

## Abstract

Female reproductive tissues repeatedly undergo controlled inflammation, immune adaptation, and tissue repair. Interleukin-33 (IL-33) and its receptor suppression of tumorigenicity 2 (ST2) sit at the center of these processes, but their roles in reproductive disease have often appeared contradictory. In this review, we integrate current evidence and propose that IL-33/ST2 signaling is not simply pro-inflammatory or anti-inflammatory. Instead, it functions as a context- and stage-dependent regulatory axis shaped by hormonal status, tissue niche, cellular targets, and disease microenvironment. Under physiological conditions, properly timed and locally restricted IL-33/ST2 activity supports ovarian tissue clearance, decidualization, embryo implantation, pregnancy maintenance, and repair. When this regulation is disrupted, the same pathway may contribute to chronic inflammation, fibrosis, immune imbalance, and reproductive dysfunction. Evidence is strongest in endometriosis and recurrent miscarriage, where experimental and mechanistic studies link IL-33/ST2 to lesion inflammation, fibrosis, ILC2 and macrophage responses, uterine receptivity, and maternal–fetal immune tolerance. In contrast, evidence in primary ovarian insufficiency and polycystic ovary syndrome remains mainly associative, involving altered serum or follicular-fluid IL-33/ST2-related markers and inflammatory-metabolic phenotypes. This evidence hierarchy argues against interpreting IL-33 as a static biomarker or a uniformly harmful mediator. Future studies should define the temporal dynamics, cellular sources, and tissue-specific targets of IL-33/ST2 signaling. Therapeutic development should prioritize precise, stage-specific, and cell-selective modulation rather than indiscriminate systemic blockade.

## 1 Introduction

Interleukin-33 (IL-33) is a member of the interleukin-1 cytokine family and was first identified in human vascular endothelial cells in 2003, where it was originally described as nuclear factor of high endothelial venules (Baekkevold et al., 2003). Its receptor, suppression of tumorigenicity 2 (ST2; also termed IL-1RL1), is the specific receptor for IL-33 and exists mainly as two isoforms: the membrane-bound signaling receptor ST2L and the soluble decoy receptor sST2, which negatively regulates IL-33 activity (Tominaga et al., 1989; Dinarello, 2005; Schmitz et al., 2005; Hayakawa et al., 2007; Sheng et al., 2025).

IL-33 is composed of 270 amino acids and exists in two principal forms: nuclear IL-33 (nIL-33) and cytokine IL-33 (cIL-33). These forms of IL-33 differ in structure, function and regulatory mechanisms (Baekkevold et al., 2003; Schmitz et al., 2005). From a structural perspective, nIL-33 contains an N-terminal nuclear/chromatin-associated region and a C-terminal cytokine-like domain responsible for binding the membrane receptor ST2L. It also has a central domain that can be cleaved by inflammatory proteases. Upon proteolytic processing, nIL-33 is converted into the cleaved form, cIL-33, which exhibits a 10 to 30-fold increase in biological activity (Onda et al., 1999; Roussel et al., 2008). IL-33 is a typical dual-function cytokine (Cayrol and Girard, 2018). Under homeostatic conditions, nIL-33 is predominantly retained in the nucleus, where it binds chromatin and contributes to transcriptional repression (Carriere et al., 2006; Roussel et al., 2008). In contrast to its nuclear role, IL-33 can be rapidly mobilized in response to cellular damage or stress. Then, it is released into extracellular milieu, where it functions as an endogenous danger-associated alarmin signal (Liew et al., 2016; Cayrol and Girard, 2018; 2022). The extracellular form of IL-33 binds ST2L to activate downstream signaling cascades, including MyD88, IRAKs, TRAF6, MAPK, and NF-κB. These pathways collectively modulate immune responses (Liew et al., 2016; He et al., 2024; Sheng et al., 2025).

A key feature of IL-33 biology is that its function is highly context dependent. IL-33 does not act as a uniformly pro-inflammatory or anti-inflammatory mediator. IL-33 biological effects are shaped by tissue type, cellular targets, physiological stage, and the duration and intensity of signaling (Liew et al., 2016; Cayrol and Girard, 2018; 2022). In some settings, transient IL-33 signaling promotes inflammatory resolution, immune tolerance, and tissue repair by enhancing the activity of ST2^+^ regulatory T cells (Tregs), type 2 innate lymphoid cells (ILC2s), and repair-associated macrophages (Gajardo Carrasco et al., 2015; Miller et al., 2017; 2021; Sugahara et al., 2022; Chen et al., 2024). In other settings, persistent or dysregulated IL-33/ST2 activation may amplify chronic inflammation, type 2 immune deviation, and fibrotic remodeling (McHedlidze et al., 2013; Weiskirchen and Tacke, 2017; Kotsiou et al., 2018; Tan et al., 2021; Yu et al., 2024). This context-dependent principle is especially relevant to female reproductive tissues, which undergo cyclic inflammation, rapid tissue breakdown and repair, and tightly regulated immune adaptation during ovulation, menstruation, implantation, and pregnancy.

Accumulating evidence indicates that IL-33/ST2 signaling is actively involved in female reproductive biology rather than being a passive inflammatory marker. In reproductive tissues, IL-33 has been detected in ovarian follicles, corpus luteum, ovarian stroma, uterine luminal and glandular epithelium, myometrium, cervix, and placental or amniotic tissues (Granne et al., 2011; Topping et al., 2013; Kulhan et al., 2019; Begum et al., 2020; Zhang et al., 2022; Lei et al., 2023). The expression of ST2 in reproductive tissues largely parallels that of IL-33. However, ST2 is rarely detected in oocytes themselves, instead being predominantly expressed in the granulosa cells surrounding IL-33^+^ oocytes within the follicle (Begum et al., 2020). Experimental studies further suggest that IL-33/ST2 signaling participates in ovarian tissue homeostasis, decidualization, embryo implantation, and maternal-fetal immune regulation. However, these effects are not uniform across tissues or disease states. Depending on the local microenvironment, IL-33 may support physiological tissue repair and immune tolerance, or alternatively contribute to chronic inflammation and fibrosis.

In this review, we focus on the physiological and pathological roles of IL-33/ST2 signaling in female reproductive tissues, integrating mechanistic insights and disease associations in conditions such as premature ovarian insufficiency (POI), polycystic ovary syndrome (PCOS), endometriosis, and recurrent miscarriage (RM). By synthesizing current evidence, we organize the literature around a unified concept: the local microenvironment and reproductive stage determine the outcome of IL-33/ST2 signaling. By applying this framework, we aim to clarify how IL-33 coordinates inflammatory activation, immune tolerance, and tissue remodeling in the ovary, endometrium, and pregnancy-related tissues under both physiological and pathological conditions. We also highlight current gaps in evidence and discuss why future translational strategies should favor precise, context-dependent modulation rather than indiscriminate systemic blockade.

## 2 Physiological role of IL-33/ST2 signaling in female reproductive tissues and pregnancy

**FIGURE 1:**
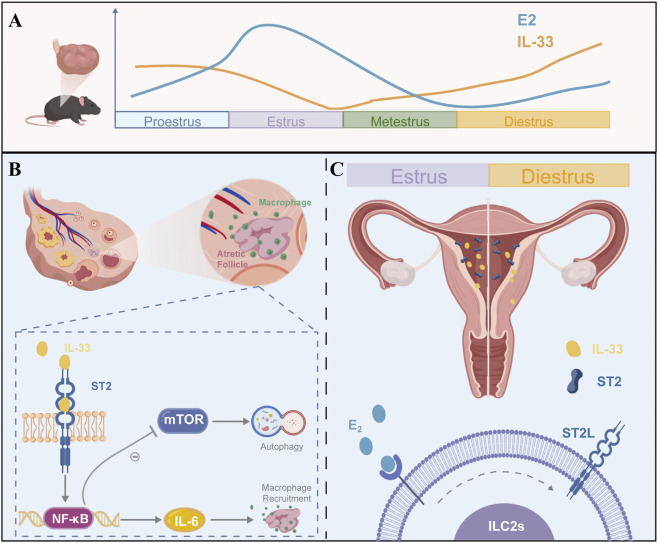
Physiological expression and regulation of IL-33/ST2 signaling in the murine ovary and uterus. **(A)** In mouse ovaries, IL-33 mRNA expression is dynamically associated with E2 levels throughout the estrous cycle, and reaches its peak during diestrus. **(B)** In the murine ovary, IL-33 primarily regulates post-ovulatory tissue clearance. IL-33 activates the NF-κB signaling pathway, which in turn suppresses mTOR expression, thereby promoting autophagy following ovulatory events. Simultaneously, enhancement of the NF-κB signaling pathway also increases IL-6 expression, facilitating macrophage recruitment to the ovary. Together, these processes promote efficient clearance after ovulation and contribute to the maintenance of ovarian microenvironmental homeostasis. **(C)** In the uterus, IL-33 expression is regulated by estrogen and varies across the estrous cycle. IL-33 expression is increasing during estrus, predominantly localized in the endometrial and glandular epithelium, whereas during diestrus, IL-33 expression is markedly reduced and primarily detected in the myometrium. In contrast, ST2 expression in the murine uterus remains relatively stable throughout the estrous cycle and is broadly distributed across uterine tissue layers. Concurrently, estrogen promotes the maintenance and expansion of uterine ILC2s, thereby enhancing IL-33/ST2-mediated immune responses.

At present, most evidence regarding IL-33/ST2 signaling in female reproductive tissues comes from studies of the mouse ovary and uterus (Ci et al., 2014; Begum et al., 2020). However, the cell-type-specific expression patterns and regulatory mechanisms of this pathway in other reproductive tissues remain incompletely understood. Available studies indicate that IL-33/ST2 expression in the ovary and uterus is sensitive to hormonal fluctuations (Ci et al., 2014; Bartemes et al., 2018; Begum et al., 2020). Moreover, IL-33/ST2 activity has been linked to immune regulation and coordinated tissue remodeling during key reproductive events (Wu et al., 2015; Wu et al., 2024; Bartemes et al., 2018). The following sections summarize the expression of IL-33/ST2 in the ovary, uterus, and pregnancy-related tissues (Table 1, 2), together with their potential physiological roles (Figure 1).

**TABLE 1:** Physiological expression of IL-IL-33/ST2 in the ovaries and uterus.

Organ	Tissue/Cell type	Molecule	Prepubertal	Sexually mature		Species	References
Ovary	Oocytes	IL-IL-33	++	++		Mouse	Begum et al. (2020)
	Granulosa cells	ST2	++	++			
	Corpus luteum	IL-IL-33	−	+++			
		ST2	−	+			
	Stromal cells	IL-IL-33	−	++			
		ST2	+	+			
	Theca layer	IL-IL-33	−	+++			
		ST2	−	+			
Uterus	LE/GE	IL-IL-33	LE+/GE−	**Estrus**	**Diestrus**		
				+++	+		
		ST2	+++	+++	+++		
	Myometrium	IL-IL-33	+	+	+++		
		ST2	+	+	+		
	Cervical tissue	IL-IL-33	—	++		Human	Kulhan et al. (2019), Zhang et al. (2022)

Expression levels are presented as semi-quantitative summaries based on the cited studies.

“−”, no detectable expression. “+”, low expression. “++”, moderate expression. “+++”, high expression. “/”, not clearly defined or not specified in the cited studies. LE, luminal epithelium; GE, glandular epithelium.

**TABLE 2:** Physiological expression of IL-IL-33/ST2 in the fetal appendages.

Organ	Tissue		Molecule	Expression	Species	References
Placenta	Chorionic plate and Chorionic villi and Basal plate vessels		IL-33	+++ nuclear	Human	Granne et al. (2011), Topping et al. (2013)
			ST2	—		
	Villous stroma		IL-33	+++ nuclear		
			ST2	—		
	Syncytiotrophoblast		IL-33	−		
			ST2	++		
Fetal membrane	Amnion	Mesenchymal fibroblasts	IL-33	+++		Lei et al. (2023)
			ST2	+++		
		epithelial cells	IL-33	+		
			ST2	+++		
	Macrophage		IL-33	++ nuclear		
			ST2	—		
Umbilical cord	Wharton’s jelly		IL-33	++ cytoplasmic		Topping et al. (2013)
			ST2	—		
	Umbilical vessels		IL-33	++ nuclear		
			ST2	—		

Expression levels and localization patterns are presented as semi-quantitative summaries based on the cited studies.

“−”, no detectable expression. “+”, low expression. “++”, moderate expression. “+++”, high expression. “/”, not clearly defined or not specified in the cited studies. Nuclear, nuclear localization. cytoplasmic, cytoplasmic localization.

### 2.1 Physiological role of IL-33/ST2 in the ovary

Under physiological conditions, IL-33 and ST2 are constitutively expressed in the mouse ovary, with dynamic spatial and temporal patterns across ovarian development and cyclic remodeling (Ci et al., 2014; Wu et al., 2015; Begum et al., 2020). In humans, IL-33 protein has been detected in follicular fluid from healthy reproductive-age women, supporting its presence in the human ovarian microenvironment, although functional evidence in humans remains limited (Tang et al., 2024). Collectively, current data suggest that ovarian IL-33/ST2 signaling is dynamically engaged during cyclic remodeling and is most strongly linked to follicular atresia-associated tissue clearance rather than to the initiation of ovulation itself.

In untreated ovaries from reproductively mature mice, immunofluorescence analysis demonstrated broad IL-33 and ST2 staining in the ovarian stroma, corpora lutea, follicles at multiple developmental stages, and the theca layer. At the follicular level, IL-33 staining was observed in oocytes, whereas ST2 staining was primarily localized to granulosa cells surrounding these IL-33^+^ oocytes. In contrast, pubescent mouse ovaries exhibited little to no detectable IL-33 or ST2 staining in the theca region or ovarian stroma (Begum et al., 2020) (Table 1). However, the identity of the dominant IL-33 producing cell types in the ovary remains controversial. One study identified a substantial proportion of IL-33^+^ cells as CD31^+^ endothelial cells (Ci et al., 2014), whereas another did not detect co-localization between IL-33 and CD31 (Begum et al., 2020). Therefore, current evidence does not yet support a definitive assignment of the dominant ovarian IL-33-producing cell type, and apparent discrepancies across studies may reflect methodological differences as much as biological variation.

Ovarian IL-33 mRNA expression is broadly maintained across mice of different reproductive ages, showing no significant age-dependent differences (Wu J. et al., 2024). Ovarian IL-33 expression exhibits pronounced temporal dynamics at both the mRNA and protein levels during superovulation. In a mouse hCG-induced ovulation model, IL-33 mRNA levels increase rapidly and peak at approximately 5 h post-injection (Ci et al., 2014). Expression of its receptor, ST2, also increases, with mRNA levels peaking at approximately 6 h after hCG injection, slightly later than the peak of IL-33, and remaining relatively elevated thereafter (Wu J. et al., 2024). Western blot analysis further indicates that in the same model, nIL-33 protein levels reach their maximum at 6 h post-hCG injection, whereas the cleaved, highly active cytokine form (cIL-33) peaks later, at approximately 9 h (Ci et al., 2014). In addition, DNA microarray analysis indicates that IL-33 is the most highly upregulated immune-related gene at 6 h after hCG administration, while ST2 ranks among the top upregulated genes (Ci et al., 2014). The above results indicate that this signaling pathway exhibits stage-specific activation during ovarian tissue remodeling in the preovulatory phase.

Consistently, under physiological conditions, IL-33 expression also varies across the estrous cycle in mice (Ci et al., 2014; Begum et al., 2020). At the mRNA level, IL-33 peaks during interestrus and declines to its lowest levels during estrus. In parallel, immunofluorescence analysis shows that the number of nIL-33^+^ cells is highest during proestrus and lowest during estrus (Ci et al., 2014) (Figure 1). In contrast, Begum et al., using a similar immunofluorescence-based approach, did not detect apparent estrous cycle–dependent changes in IL-33 or ST2 expression within follicles or corpora lutea (Begum et al., 2020). This discrepancy may reflect differences in antibody specificity, detection of nuclear *versus* total IL-33, and tissue sampling strategies, particularly the lack of systematic assessment of stromal compartments in some studies.

Functional studies most strongly support a role for IL-33 in coordinating follicular atresia-associated clearance, including macrophage recruitment, autophagy-related processes, and local inflammatory signaling. In mouse ovaries, follicular atresia is accompanied by increased cIL-33 levels and a surge in macrophage infiltration (Ci et al., 2014). Evidence from IL-33−/− mouse models indicates that IL-33 contributes to the clearance of atretic follicles. Loss of IL-33 leads to reduced macrophage infiltration and impaired autophagic activity, accompanied by increased mTOR expression and decreased IL-6 levels (Wu et al., 2015). These alterations result in defective removal of degenerating follicles and accelerated depletion of ovarian reserve. In addition, ST2 expression has been detected in granulosa cells of early atretic follicles, and IL-33 deficiency or blockade reduces NF-κB signaling and the expression of inflammatory mediators, including IL-6 (Wu J. et al., 2024), supporting a role for the IL-33 ST2 NF-κB axis in coordinating macrophage recruitment and tissue remodeling.

Overall, current evidence suggests that IL-33 primarily contributes to inflammation resolution and tissue remodeling during follicular atresia rather than acting as a direct initiator of ovulation or follicular degeneration (Wu et al., 2015; Wu J. et al., 2024). However, most available data are derived from mouse models, and the relevance of these findings to human ovarian physiology remains unclear. In addition, the precise cellular sources of IL-33 and the mechanisms linking its nuclear and cleaved forms to distinct functional outcomes require further investigation.

### 2.2 Physiological role of IL-33/ST2 in the uterus

IL-33 is expressed in the mouse uterus, and its expression level and spatial distribution are influenced by the estrous cycle. In contrast, ST2 expression in the uterus does not depend on the estrous cycle. In prepubertal mice, IL-33^+^ cells are absent in the uterine glandular epithelium, and the protein expression of IL-33/ST2 in the luminal epithelium and myometrium is low (Begum et al., 2020) (Table 1). In immunofluorescence sections of sexually mature mice, IL-33^+^ cells are highly expressed during estrus, predominantly localized in the luminal and glandular epithelium, while during diestrus, they accumulate in the myometrium. Additionally, ST2^+^ cells are mainly found in the luminal and glandular epithelium throughout the estrous cycle, with a small proportion in the myometrium (Begum et al., 2020) (Figure 1). This suggests that IL-33 plays a stage-specific role in the uterus. These changes in IL-33 expression may be related to fluctuations in hormone levels during the estrous cycle.

However, it is noteworthy that the protein level of IL-33 in the uterus of estrogen-supplemented mice did not exhibit significant fluctuations (Bartemes et al., 2018), whereas IL-33 mRNA expression in the uterus significantly increased after superovulation (Begum et al., 2020). This indicates that the expression of IL-33 in the uterus may be regulated by hormones other than estrogen during the estrous cycle.

The functional role of IL-33 in the uterus extends beyond its regulation of expression. Studies have shown that IL-33 interacts with innate lymphoid cells type 2 (ILC2s) in the uterine immune system. ST2^+^ ILC2s are present in the mouse uterus, and evidence indicates that IL-33 can activate these cells both *in vitro* and *in vivo* (Bartemes et al., 2018). Interestingly, ovariectomy significantly reduces the number of ST2^+^ ILC2s in the uterus, but this reduction can be restored by exogenous estrogen supplementation without affecting the level of IL-33 protein in the uterus (Bartemes et al., 2018). This suggests that estrogen amplifies the IL-33/ST2 signaling pathway in the uterus by activating and expanding ST2^+^ ILC2s, which may influence immune responses and tissue remodeling during the estrous cycle.

Moreover, research has shown that ST2-deficient female mice do not exhibit significant differences in the number of offspring compared to wild-type mice. However, the mortality rate of their pups within 24 h of birth is significantly higher, and this phenotype is independent of the male mouse MHC haplotype (Bartemes et al., 2018). This suggests that the IL-33/ST2 pathway plays a role in establishing a uterine environment conducive to offspring survival.

Overall, the current evidence indicates that the IL-33/ST2 signaling pathway in the uterus is regulated by hormonal fluctuations during the estrous cycle and plays a crucial role in immune modulation, particularly through its interaction with ILC2 cells. However, despite support from mouse model data, human studies remain limited. Future research should further explore the regulatory mechanisms of IL-33/ST2 signaling and its specific role in uterine immune homeostasis and reproductive health.

### 2.3 Physiological role of IL-33/ST2 in pregnancy

IL-33/ST2 is widely expressed in the human placenta, fetal membranes, and umbilical cord. IL-33 is predominantly localized to placental vascular tissue, fetal membrane cells, and umbilical cord mesenchymal cells. Amniotic fibroblasts represent the major cellular source of IL-33/ST2, whereas ST2 is distributed in the placental syncytiotrophoblast and fetal membrane cells (Table 2). Notably, amniotic IL-33/ST2 expression is significantly upregulated at the onset of labor (Granne et al., 2011; Topping et al., 2013; Lei et al., 2023). IL-33/ST2 signaling plays a central role in early pregnancy by regulating immune responses and mediating tissue remodeling. IL-33 is expressed by diverse uterine cell types and is under tight spatiotemporal regulation throughout gestation.

In mice, uterine IL-33 expression is markedly upregulated after implantation. Myometrial fibroblasts and myofibroblasts serve as the primary cellular sources of IL-33, and decidual endothelial IL-33 expression peaks at embryonic day 7.5 (E7.5) (Valero-Pacheco et al., 2022). Secreted IL-33 acts on ST2-expressing immune cells, including regulatory T cells, ILC2s, and M2-like macrophages, to coordinate type 2 immune responses and tissue remodeling, thereby sustaining the uterine structural remodeling required for successful pregnancy (Valero-Pacheco et al., 2022). IL-33 knockout (KO) dams exhibit defective uterine tissue remodeling and impaired type 2 immunity, resulting in adverse pregnancy outcomes including increased embryo resorption and fetal growth restriction (Valero-Pacheco et al., 2022). These findings confirm that the IL-33/ST2 axis acts as a master regulator of uterine type 2 immune responses in early pregnancy and is indispensable for uterine structural remodeling and the establishment of a maternal-fetal immune-tolerant microenvironment.

IL-33 functions in a highly context-dependent manner. During early pregnancy, IL-33 supports gestation by maintaining the functional maturation of ILC2s and M2-like macrophages. Conversely, dysregulated or uncontrolled IL-33/ST2 signaling alters macrophage polarization and raises the risk of pregnancy complications (Valero-Pacheco et al., 2022). This reveals the dual role of IL-33: it maintains immune tolerance and tissue homeostasis in early pregnancy, yet triggers pathological effects when signaling is excessively activated or dysregulated.

The essential role of IL-33 in decidualization is well established. In IL-33 KO dams, the expression of key decidualization genes including Wnt4 and Fkbp5 is significantly reduced in the decidua. This leads to abnormal decidual angiogenesis and impaired spiral artery remodeling, which are closely associated with smaller placental size and fetal growth restriction (Valero-Pacheco et al., 2022). Thus, IL-33 sustains placental perfusion to supply essential nutrients for fetal development.

In early human pregnancy, the physiological function of IL-33/ST2 signaling is most evident in regulating decidualization and uterine receptivity. During *in vitro* decidualization of primary human endometrial stromal cells (HESCs), the IL-33/ST2 axis is activated sequentially: IL-33 expression and secretion rise transiently in early decidualization, ST2L is induced early, and sST2 becomes dominant in the late stage. This dynamic pattern indicates that IL-33/ST2 signaling is dynamically regulated during decidualization rather than being constitutively activated (Salker et al., 2012).

Immunohistochemical analysis of human endometrial biopsies reveals prominent IL-33 staining in stromal cells, especially in decidualized stromal cells in the mid-secretory phase, further confirming its physiological relevance in the human uterus (Salker et al., 2012). Functional experiments demonstrate that HESCs with IL-33 or ST2L knockdown fail to maintain the uterine microenvironment supportive of embryo implantation (Salker et al., 2012), indicating that IL-33/ST2 signaling is crucial for sustaining normal uterine receptivity and promoting decidualization. Furthermore, the upregulation of sST2 in late decidualization confirms the role of this axis in the timely closure of the uterine receptive window.

In summary, the IL-33/ST2 axis does not function as a mere pro-inflammatory factor in human pregnancy. Instead, it controls the activation and resolution of the signaling axis across gestational stages to coordinate decidualization and uterine receptivity. By orchestrating the establishment and timely termination of the receptive window, it supports the normal progression of pregnancy.

## 3 Role of IL-33/ST2 signaling in female reproductive diseases

Growing evidence supports the involvement of IL-33/ST2 signaling in the pathogenesis of multiple female reproductive disorders (Salker et al., 2012; Al-Taie et al., 2014; Karakose et al., 2016; Miller et al., 2021; Tang et al., 2024). In this section, we review and integrate current findings on the role of IL-33/ST2 axis in several female reproductive pathologies, including polycystic ovary syndrome (PCOS), POI, endometriosis, and recurrent miscarriage (RM). Table 3 and Supplementary Table S1 summarize the expression profiles and functional implications of IL-33/ST2 signaling in female reproductive pathologies, excluding malignancies of the reproductive system.

**TABLE 3:** Expression and role of the IL-33/ST2 signaling axis in female reproductive pathologies.

Reproductive pathology	Expression and role of IL-33/ST2	References
Polycystic ovarian syndrome	Serum IL-33 concentrations were markedly increased in women with PCOS compared with healthy controls.	Al-Taie et al. (2014), Karakose et al. (2016)
	Serum IL-33 concentrations in PCOS patients are associated with glucose metabolism and antioxidant capacity.	(Al-Taie et al., 2014; D Prabhu and Valsala Gopalakrishnan, 2020)
	PCOS patients with higher circulating IL-33 levels also exhibited increased low-density lipoprotein (LDL) levels.	Karakose et al. (2016)
	Serum IL-33 concentrations in PCOS patients are associated with inflammatory response and immune regulation.	(Karakose et al., 2016; D Prabhu and Valsala Gopalakrishnan, 2020)
Primary ovarian insufficiency	Elevated IL-33 levels in FF were associated with POI, whereas sST2 levels showed a negative correlation with POI progression.	Tang et al. (2024)
Endometriosis	Elevated IL-33 levels in both plasma and PF were associated with endometriosis, and the magnitude of IL-33 increase correlated positively with disease severity.	(Chacho et al., 1986; Santulli et al., 2012; Itoh et al., 2013; Mbarik et al., 2015; Miller et al., 2017 ; 2021)
	sST 2 levels were higher in PF than in healthy women, and the extent of this increase correlated with the severity of endometriosis.	Mbarik et al. (2015)
	IL-33 induces ILC 2 to stimulate the inflammatory response to drive endometriosis.	(Miller et al., 2021; Sugahara et al., 2022)
	IL-33 promotes tissue fibrosis by promoting peritoneal macrophage polarization to the M2 phenotype and accelerates the progression of endometriosis.	Ono et al. (2020)
	IL-33 is involved in endometriosis by stimulating inflammatory response, angiogenesis and lesion proliferation.	Miller et al. (2017)
Recurrent miscarriage	Certain IL-33 gene SNPs are associated with RM susceptibility, while other SNPs show no significant association.	(Yue et al., 2016; Zidan et al., 2018; Soheilyfar et al., 2019)
	Decreased serum IL-33 and Foxp3 levels are associated with recurrent miscarriage (RM).	(Mei et al., 2010; Yue et al., 2016; Zidan et al., 2018)
	Altered sequential activation of the IL-33/ST2L/sST2 axis in HESCs is linked to RM.	Salker et al. (2012)
	Elevated IL-33 levels and coordinated activation of the IL-33/sST2 axis contribute to embryo implantation and the maintenance of maternal immune tolerance toward the fetus.	(Mei et al., 2010; Kaitu’u-Lino et al., 2012; Salker et al., 2012; La Rocca et al., 2014; Gajardo Carrasco et al., 2015; Gajardo et al., 2015; Yue et al., 2016; Zidan et al., 2018)

### 3.1 Polycystic ovary syndrome

Polycystic ovary syndrome (PCOS) is one of the most prevalent common disorders in women of reproductive age, with an estimated prevalence of approximately 6%–13% (Mousa et al., 2023). In addition to reproductive symptoms such as menstrual irregularities, hyperandrogenism, and infertility, PCOS is frequently associated with metabolic abnormalities, including insulin resistance and impaired glucose tolerance (Anagnostis et al., 2021; Mousa et al., 2023; Belsti et al., 2024). Chronic low-grade inflammation is also commonly observed in PCOS patients (Wang et al., 2023). Persistent inflammation may promote hyperandrogenism independently of obesity, creating a self-reinforcing cycle that exacerbates the progression of the disease (Deng et al., 2024; De Robles et al., 2025). Against this background, IL-33/ST2 has attracted attention as a potential mediator linking inflammation and metabolic disturbances in PCOS. However, most of the existing evidence comes from associative clinical studies, and direct mechanistic support remains limited.

Several studies have reported significantly elevated serum IL-33 levels in PCOS patients compared to healthy controls (Al-Taie et al., 2014; Karakose et al., 2016). Furthermore, IL-33 has been found to positively correlate with several metabolic and inflammatory markers, including glycated hemoglobin (HbA1c), low-density lipoprotein cholesterol (LDL-C), and IL-17A (Al-Taie et al., 2014; Karakose et al., 2016). These findings support an association between IL-33-related markers and the inflammatory-metabolic phenotype of PCOS, but they do not establish a causal role for IL-33/ST2 signaling in the pathogenesis of the disease.

Despite limited experimental evidence, IL-33’s involvement in the inflammatory processes of PCOS is supported by data from animal models. In a DHEA-induced mouse model of PCOS, γ-linolenic acid (GLA) was shown to reduce the expression of IL-33 and TNF-α in the ovaries, as well as improve inflammation and androgen-related phenotypic features (D Prabhu and Valsala Gopalakrishnan, 2020). This finding supports the idea that IL-33 plays a role in ovarian inflammation in PCOS-like states, but it does not prove that IL-33 is a causal factor in the disease mechanism.

Moreover, studies have reported that serum IL-33 levels are positively correlated with LDL-C in PCOS patients, further indicating that IL-33 may be involved in metabolic networks within PCOS (Karakose et al., 2016). Notably, the relationship between IL-33 and metabolic features is not consistent across different subgroups of PCOS patients. One study demonstrated that, when stratified by the presence of diabetes and insulin resistance, the correlation between serum IL-33 and oxidative stress, antioxidant capacity, and insulin resistance varied significantly (Al-Taie et al., 2014). Specifically, in individuals without insulin resistance or type 2 diabetes, IL-33 was positively correlated with the homeostasis model assessment of insulin resistance (HOMA-IR) and negatively correlated with oxidative stress. In contrast, in PCOS patients with type 2 diabetes and insulin resistance, IL-33 was negatively correlated with HOMA-IR and positively correlated with oxidative stress and antioxidant capacity (Al-Taie et al., 2014). These inconsistent patterns do not suggest a simple linear relationship, but rather may indicate that IL-33 reflects different biological states of PCOS, such as metabolic subtypes, disease stage, or immune-metabolic adaptation in different microenvironments. However, this interpretation remains speculative and requires further validation.

Overall, current evidence suggests that changes in IL-33/ST2-related markers are associated with inflammation, oxidative stress, and metabolic abnormalities in PCOS. However, the available data are still insufficient to define a clear tissue-specific or cell-specific mechanism. In particular, it remains unclear whether IL-33/ST2 is a causal factor in the pathogenesis of PCOS or mainly reflects the heterogeneity of the disease. Therefore, future research should focus on ovarian tissue-level analysis, cell-specific mechanisms, and phenotype-stratified cohort studies in order to reliably assess the translational significance of this pathway.

### 3.2 Primary ovarian insufficiency

POI is a clinical syndrome marked by the reduction of ovarian function prior to the age of 40. The primary symptoms of POI involve irregular menstruation, increased gonadotropin levels, and reduced estrogen levels (Nash and Davies, 2024; Panay et al., 2024). Because aging is a major driver of chronic disease and health decline, the impact of POI extends beyond fertility and quality of life. POI is associated with increased long-term risks, including osteoporosis and fracture, cardiovascular disease, and adverse cognitive outcomes, and may ultimately reduce life expectancy if left untreated (Guo et al., 2022; Panay et al., 2024).

Recent studies have shown that basal inflammation is a key characteristic of aging (Ferrucci et al., 2005; Pilling et al., 2015). Although many studies have shown that there is a chronic inflammatory environment in patients with POI, no specific inflammatory markers for POI have been identified to date (Huang et al., 2019; Yang et al., 2020; Tang et al., 2023; Zavatta et al., 2023). Recent evidence indicates a significant increase in IL-33 levels in the FF of patients with POI compared with healthy controls. Elevated FF IL-33 levels were positively correlated with serum follicle-stimulating hormone (FSH) and luteinizing hormone (LH). In contrast, IL-33 levels showed negative correlations with anti-Müllerian hormone (AMH), estradiol (E2), and testosterone (T). Additionally, as POI progressed, the levels of sST2 in FF decreased, suggesting activation of the IL-33/ST2 axis, which may enhance IL-33’s role in the progression of POI. However, in the serum of most POI and control group patients, IL-33 levels were undetectable, indicating a tissue-specific connection between ovarian failure and increased IL-33 levels in FF (Tang et al., 2024). These findings suggest that activation of the IL-33/ST2 signaling pathway is involved in POI progression. However, it remains unclear whether IL-33 is a causative factor in POI or merely reflects disease progression, and its precise mechanisms and effector cells are still undefined.

A Mendelian randomization study has identified IL-33 as a risk factor for POI, with higher serum IL-33 levels being associated with an increased risk of the condition (Wu et al., 2025), suggesting that IL-33 may be a potential risk factor for POI rather than merely a disease-associated phenomenon. Additionally, research has shown that in women undergoing *in vitro* fertilization or intracytoplasmic sperm injection, higher FF sST2 levels were correlated with larger follicles and better embryo quality (Southcombe et al., 2013). Conversely, FF IL-33 levels were negatively associated with the rate of high-quality embryos on Day 3 of embryo culture (Tang et al., 2024). These findings indicate that sST2 and IL-33 levels in FF reflect the degree of follicular development and can serve as indicators for predicting ovarian functional reserve and embryonic quality in POI patients. These results support the hypothesis that IL-33 contributes to ovarian aging and influences follicular quality, though further research is required to elucidate the underlying mechanisms underlying this association.

The ovary is unique in that it experiences functional aging earlier than other organs, making ovarian dysfunction a sentinel event in the trajectory of female aging (Yang et al., 2024). The role of IL-33 in ovarian aging has not been fully elucidated, and existing evidence is largely based on correlative studies. Ovarian aging is associated with the progressive activation of inflammatory pathways, placing the ovary in a state of sterile chronic inflammation (Ben Yaakov et al., 2023; Isola et al., 2024; Wu M. et al., 2024). Additionally, in Treg cells, FOXP1 interacts with FOXP3 at genomic loci in the IL-33 signaling pathway, co-regulating the immunosuppressive function of Treg cells (Konopacki et al., 2019). Disruptions in the regulatory relationship between IL-33 and FOXP1 may contribute to sterile chronic inflammation in aging ovaries and POI patients (Briley et al., 2016; Ansere et al., 2021; Lliberos et al., 2021). However, these mechanisms remain speculative and require further investigation. Understanding the exact role of FOXP1 and IL-33 in immune regulation within the ovary could become a major focus of future research.

In conclusion, although increasing evidence suggests a correlation between IL-33/ST2 and the progression of POI, the direct causal relationship remains unclear. The current data are mostly associative, and more mechanistic studies are needed to determine whether IL-33 is a modifiable factor in POI. Future research should focus on elucidating the regulatory mechanisms of IL-33 in the ovary, particularly its relationship with immune responses and cellular aging processes, to assess its potential as a therapeutic target for POI.

### 3.3 Endometriosis

Endometriosis is a chronic inflammatory disease characterized by the growth of endometrial-like tissue outside the uterus. It affects approximately 10%–15% of women of reproductive age (de Ziegler et al., 2010). Its main symptoms include dysmenorrhea, irregular menstruation, dyspareunia, and infertility, which markedly impair quality of life (Zondervan et al., 2020). Endometriosis is commonly classified into ovarian endometriosis (OMA), superficial peritoneal endometriosis (SUP), and deep infiltrating endometriosis (DIE). Among these subtypes, DIE is the most severe and is associated with ectopic endometrial proliferation, marked inflammation, and fibrotic remodeling (Anaf et al., 2000; Vicino et al., 2009).

Endometriosis is closely associated with immune dysregulation. Several studies suggest that the disease shows a bias toward type 2 immune responses (Podgaec et al., 2007; May et al., 2010). IL-33 is thought to participate in immune regulation and fibrosis, both of which are consistent with the characteristic of endometriosis. (Santulli et al., 2012; Di Carmine et al., 2022). Compared with healthy controls, women with endometriosis show significantly higher IL-33 levels in plasma and peritoneal fluid (PF), especially in cases of DIE (Santulli et al., 2012; Itoh et al., 2013; Mbarik et al., 2015; Miller et al., 2021). These findings suggest that IL-33 may be involved in the development of endometriosis.

Experimental studies provide further support for this association. In mouse models of endometriosis, IL-33 gene knockout or IL-33 neutralization by sST2 can inhibit downstream inflammatory signal transduction, such as IRAK4 in the MyD88/NF-κB pathway all reduce lesion size (Kato et al., 2019). In contrast, IL-33 supplementation activates inflammation, angiogenesis, and lesion proliferation. It also increases immune cell recruitment to the peritoneal cavity and lesion microenvironment, thereby aggravating disease progression in mice (Miller et al., 2017). In addition, ROS, TGF-β1, and high estrogen levels in the ectopic microenvironment can induce IL-33 secretion from human ectopic endometrial stromal cells *in vitro*. IL-33 then activates the PKA pathway and induces incomplete epithelial-mesenchymal transition (EMT) through β-catenin, ultimately accelerating lesion fibrosis in a mouse model of endometriosis (Ruan et al., 2024). These findings suggest that IL-33 may promote endometriosis progression through two related mechanisms: ST2-dependent activation of NF-κB signaling, which promotes inflammation and angiogenesis, and PKA-dependent Wnt/β-catenin signaling, which promotes EMT and fibrosis.

ILC2s are present in the PF of women with endometriosis, and their numbers are increased in ovarian endometriotic cysts and ectopic lesions compared with normal endometrium (Miller et al., 2017; 2021). In a mouse model of endometriosis, transcriptomic analysis of peritoneal lavage after IL-33 supplementation showed that ST2^+^ ILC2s were the only ST2^+^ immune cell population that increased significantly (Miller et al., 2021). Further experiments showed that, after rIL-33 supplementation, lesion growth and fibrosis were reduced in ILC2-deficient and ILC2-depleted mice (Miller et al., 2021). These data indicate that IL-33/ST2-driven endometriosis pathology is strongly dependent on ILC2s in experimental models. To further explore the source of increased ST2^+^ ILC2s in PF and lesions, studies have shown that women with endometriosis have fewer ILC2s in their eutopic endometrium than healthy women. In contrast, the proportion of ILC2s is increased in endometriotic lesions, while the number and proportion of ILC2s in peripheral blood remain unchanged (Miller et al., 2017; 2021; Sugahara et al., 2022). These findings suggest that IL-33 may mainly act as a local microenvironmental factor within ectopic lesions. It may promote the recruitment, retention, or expansion of ILC2s near lesions rather than inducing systemic ILC2 expansion. This local effect may further enhance inflammation, angiogenesis, and lesion proliferation. Therefore, more detailed phenotypic and functional analyses of endometrial and lesion-associated ILC2 subsets are needed to clarify how IL-33 sustains chronic inflammation in endometriosis.

Macrophages are another important cellular target of IL-33 in endometriosis. IL-33 can stimulate macrophages to secrete pro-inflammatory cytokines, induces nerve growth within lesions, as well as co-localization of macrophages and nerve fibers (Li et al., 2025). These findings suggest a direct role for IL-33-mediated neuroimmune interaction in endometriosis-associated chronic pain. This mechanism highlights the dual role of IL-33 in immune regulation and pain generation.

Other studies have shown that peritoneal macrophages are increased in women with endometriosis (Bacci et al., 2009; Itoh et al., 2013; Borrelli et al., 2014; Miller et al., 2021). *In vitro*, IL-33 can stimulate primary macrophages from PF to polarize toward an M2 phenotype through ST2. These polarized M2 macrophages secrete IL-1β and further promote IL-33 secretion from endometriotic lesion cells through the p38 MAPK pathway. This forms an IL-33/IL-1β positive feedback loop that accelerates lesion growth (Ono et al., 2020). In addition, IL-33 can regulate lesion cell survival through macrophage activation. In an *in vitro* study using human primary endometriotic cells, IL-33 inhibited ferroptosis through the p38/JNK–ATF3–SLC7A11 axis, thereby protecting ectopic endometrial cells from damage in an iron-overloaded microenvironment and supporting lesion persistence (Wu et al., 2023). More recently, *in vitro* experiments showed that IL-33 secreted by pro-endometriotic macrophages mainly promoted lesion formation and proliferation during the early stage of disease, whereas at later stages it helped maintain lesion stability and drove pain. Its expression was also positively associated with lesion gland number and the degree of fibrosis (Fattori et al., 2025). Together, these findings suggest that IL-33 not only regulates immune cell polarization, but also supports lesion cell survival, inflammatory persistence, fibrosis, and pain in the endometriotic microenvironment.

In short, evidence from clinical observations, *in vitro* mechanism studies and animal models all confirms the key role of the IL-33/ST2 axis in endometriosis. However, there are still two key problems unsolved. First, it is the spatiotemporal-specific regulation mechanism of IL-33 in different disease stages. In the early stage of the disease, IL-33 drives inflammatory outburst through ILC2. In the late stage, it maintains fibrosis and chronic pain through macrophage/nerve interaction. The molecular switch mechanism of this biphasic effect is not clear yet. Second, it is the relative contribution of IL-33 from different cell types (such as stromal cells and macrophages). In the future, therapies targeting IL-33 are expected to relieve inflammation, fibrosis and pain related to endometriosis, providing a new and promising direction for the clinical treatment of this disease.

### 3.4 Recurrent miscarriage

RM is commonly defined as two or more pregnancy losses, usually referring to clinically recognized pregnancy losses before 20–24 weeks of gestation (Dimitriadis et al., 2020). RM affects approximately 1%–2% of couples and represents an important reproductive health burden for women attempting to conceive (Dimitriadis et al., 2020; ESHRE Guideline Group on RPL et al., 2023). The etiology of RM is highly heterogeneous and may involve uterine anomalies, endocrine and metabolic disorders, parental or embryonic chromosomal abnormalities, thrombophilia, infections, lifestyle-related factors, genetic susceptibility, and immune dysregulation (Tise and Byers, 2021; ESHRE Guideline Group on RPL et al., 2023). Despite systematic evaluation, approximately 40%–50% of patients are still classified as having unexplained RM, highlighting persistent gaps in mechanism-based diagnosis and patient stratification (ESHRE Guideline Group on RPL et al., 2023; Yu et al., 2023).

Current evidence suggests that IL-33/ST2 signaling is involved in early pregnancy, especially in decidualization, embryo implantation, and the establishment of maternal–fetal immune tolerance (Mei et al., 2010; Salker et al., 2012; Yue et al., 2016; Zidan et al., 2018). IL-33 does not appear to function merely as a constitutive inflammatory mediator. Instead, it may act as a temporal regulator of the local decidual microenvironment. Properly timed activation of IL-33/ST2 signaling may help establish a transient inflammatory window that supports implantation. Failure to initiate or resolve this window may contribute to pregnancy failure. The clearest mechanistic evidence currently comes from studies using human endometrial stromal cells (HESCs). During normal decidualization, the IL-33/ST2L/sST2 axis in HESCs shows sequential activation. Decidualizing HESCs rapidly release IL-33 and upregulate membrane-bound ST2L together with other pro-inflammatory mediators, thereby generating a transient inflammatory microenvironment that supports embryo implantation (Salker et al., 2012). Functional experiments further showed that HESCs support embryo implantation only during this pro-inflammatory phase. Knockdown of IL-33 in decidualized HESCs markedly reduces their implantation-supportive capacity (Salker et al., 2012). In contrast, HESCs derived from patients with RM show disrupted sequential activation of the IL-33/ST2L/sST2 axis. These findings suggest that abnormal temporal regulation of IL-33/ST2 signaling in decidualizing stromal cells may contribute to impaired uterine receptivity in RM.

IL-33 has also been implicated in the regulation of immune tolerance at the maternal–fetal interface (Mei et al., 2010; La Rocca et al., 2014; Hu et al., 2015). Compared with normal pregnancy, decidual stromal cells from patients with RM show lower protein levels of IL-33 and ST2 (Hu et al., 2014). In normal pregnancy, IL-33 produced by decidual stromal cells may promote a shift of local immune responses toward a Th2-biased profile (Hu et al., 2015). In addition, IL-33 can activate the NF-κB pathway and downregulate granzyme A and perforin expression in decidual natural killer cells isolated from human tissues, thereby reducing their cytotoxicity and supporting maternal–fetal immune tolerance (Hu et al., 2014; 2015). On the other hand, patients with RM often show reduced proportions of CD4^+^ CD25^high^ T cells, decreased Foxp3 expression in decidual tissue, and impaired Treg number and suppressive function (Mei et al., 2010; La Rocca et al., 2014). IL-33 can promote Treg differentiation and functional adaptation in inflammatory environments, including the conversion of CD4^+^ Foxp3^−^ T cells into Foxp3-expressing Tregs (Gajardo et al., 2015). These findings support the possibility that IL-33-responsive immune regulation contributes to local maternal–fetal tolerance. However, the specific contribution of IL-33-mediated T-cell regulation to RM remains unclear.

Clinical observations also support an association between altered IL-33 signaling and RM, but these findings should be interpreted cautiously. Several studies have reported lower serum IL-33 and Foxp3 levels in patients with RM than in healthy pregnant controls (Mei et al., 2010; Yue et al., 2016; Zidan et al., 2018). These findings are consistent with the view that insufficient IL-33-mediated immune regulation may impair pregnancy maintenance. However, circulating IL-33 levels may not accurately reflect local IL-33/ST2 activity at the maternal–fetal interface. In addition, Kaitu’u-Lino et al. observed increased serum IL-33 levels in women who subsequently miscarried after 6 weeks of gestation (Kaitu’u-Lino et al., 2012). The biological significance of this increase remains unclear. It may be related to gestational timing, tissue damage, or systemic inflammatory activation, and should not be simply interpreted as evidence that elevated IL-33 is either protective or pathogenic.

## 4 Conclusions and future perspectives

Current evidence supports a working framework in which the IL-33/ST2 signaling axis should not be viewed as a simple pro-inflammatory or anti-inflammatory pathway. Instead, it appears to act as a context- and stage-dependent regulatory axis in female reproductive tissues. Its effects are shaped by reproductive stage, hormonal status, tissue compartment, cellular target, and disease microenvironment.

Under physiological conditions, appropriately timed and spatially restricted IL-33/ST2 signaling may contribute to ovarian tissue remodeling, post-ovulatory clearance, endometrial adaptation, decidualization, implantation, and early pregnancy-related immune regulation. In these settings, IL-33/ST2 signaling appears to link transient inflammation with tissue repair and immune adaptation. However, when the timing, intensity, or cellular targeting of this pathway becomes dysregulated, the same signaling axis may contribute to chronic inflammation, fibrotic remodeling, immune imbalance, and reproductive dysfunction.

Importantly, the strength of evidence differs across reproductive disorders. In endometriosis and recurrent miscarriage, experimental and mechanistic studies provide relatively stronger support for IL-33/ST2 involvement. In endometriosis, IL-33 has been linked to lesion inflammation, angiogenesis, ILC2 expansion, macrophage activation, EMT, fibrosis, ferroptosis resistance, and pain-related neuroimmune interactions. In recurrent miscarriage, evidence from HESC decidualization models and maternal–fetal immune studies supports a role for temporally regulated IL-33/ST2 signaling in uterine receptivity and immune tolerance. By contrast, evidence in PCOS and POI remains more limited and is mainly based on circulating or follicular fluid biomarkers, clinical associations, and indirect experimental observations. Therefore, IL-33/ST2 should not yet be considered a proven causal driver in these conditions.

The apparently dual effects of IL-33 in female reproductive diseases are best explained by differences in local context rather than by an intrinsic protective or pathogenic property of IL-33 itself. Altered hormonal regulation, disrupted stromal–immune communication, abnormal macrophage or ILC2 responses, impaired Treg-mediated tolerance, and disease-stage-specific changes may all shift IL-33/ST2 signaling from a homeostatic repair program toward a pathological inflammatory or fibrotic response.

Several key questions remain unresolved. First, future studies should define the temporal dynamics of IL-33/ST2 signaling across ovulation, decidualization, implantation, pregnancy, and disease progression. Static measurements of circulating IL-33 are unlikely to capture its local and stage-specific functions. Second, the cell-type-specific sources and targets of IL-33 in reproductive tissues need to be clarified, particularly in stromal cells, epithelial cells, endothelial cells, macrophages, ILC2s, dNK cells, and Tregs. Third, it will be important to determine when IL-33-mediated repair responses become persistent, fibrotic, or pathological.

From a translational perspective, these findings argue against non-selective systemic targeting of the IL-33/ST2 pathway. Because IL-33 may support normal reproductive processes such as tissue repair, implantation, and early pregnancy immune adaptation, future strategies should focus on stage-specific, tissue-restricted, or cell-selective modulation. Similarly, the value of IL-33 as a biomarker will depend on contextual interpretation, including reproductive stage, hormonal status, disease phenotype, inflammatory state, and local immune-cell composition.

Overall, IL-33/ST2 signaling is best understood as a dynamic regulatory network rather than a static inflammatory marker. Defining its context-specific functions will be essential for understanding female reproductive disease mechanisms and for developing more precise diagnostic or immunomodulatory strategies in the future.

## Glossary

AREG: amphiregulin
FSH: follicle-stimulating hormone
DSCs: decidual stromal cells
dNKs: decidual nucleated granulocytes
HIV: human immunodeficiency virus
IL-1RAcP: interleukin-1 receptor accessory protein
Th2: T helper 2 cells
AMH: anti-Müllerian hormone
CRP: C-reactive protein
CDKN1A/p21: cyclin-dependent kinase inhibitor 1A
cIL-33: cytokine IL-33
DIE: deeply infiltrating endometriosis
EMT: epithelial-mesenchymal transition
E2: estradiol
ECM: extracellular matrix
FAO: fatty acid oxidation
FF: follicular fluid
FOXP1: forkhead box P1
HbA1c: glycated hemoglobin
GVHD: graft-versus-host disease
GM-CSF: granulocyte–macrophage colony-stimulating factor
ILC2s: group 2 innate lymphoid cells
HTH: helix–turn–helixHTH
HBV: hepatitis B virus
HESCs: human endometrial stromal cells
IL-33R: IL-33 receptor
IGT: impaired glucose tolerance
IFN-γ: interferon-γ
IL-33: Interleukin 33
IRAKs: interleukin-1 receptor–associated kinases
IL-1RL1: interleukin-1 receptor-like 1
LPS: lipopolysaccharide
LH: luteinizing hormone
MAPK: mitogen-activated protein kinase
MyD88: myeloid differentiation primary response 88
NASH: nonalcoholic steatohepatitis
NF-κB: nuclear factor κB
OMA: ovarian endometriosis
OXPHOS: oxidative phosphorylation
PF: peritoneal fluid
PCOS: polycystic ovary syndrome
POI: primary ovarian insufficiency
rhIL-33: recombinant human IL-33
RM: recurrent miscarriage
Tregs: regulatory T cells
sST2: soluble form
ST2L: ST2 ligand form
SUP: superficial peritoneal endometriosis
ST2: suppression of tumorigenicity 2
T: testosterone
TIR: toll/interleukin-1 receptor
TRAF6: tumor necrosis factor receptor–associated factor 6
TNF-α: tumor necrosis factor-α
T1: type 1–like receptor
GLA: γ-linolenic acid
