# Supplementary material for: The role of IL-33/ST2 signaling in female reproductive diseases
Source: Front Cell Dev Biol. 2026 Jun 12;14:1808099. doi: 10.3389/fcell.2026.1808099 (PMC13303986; doi:10.3389/fcell.2026.1808099)
Supplement: Supplementary file 1 [file Table1.docx]

Supplementary Material

# Table S1. Expression and role of the IL-33 / ST2 signaling axis in other female reproductive pathology.

| **Reproductive pathology** | **Expression and role of IL-33 / ST2** | **Ref** |
| --- | --- | --- |
| Placenta previa accreta | The level of IL-33 is significantly increased in the serum of patients with placenta previa. | (Ozler et al., 2021) |
| Pre-eclampsia and pregnancy | PE patients have increased plasma levels of IL-33 and sST2. | (Yue et al., 2016; Zidan et al., 2018; Soheilyfar et al., 2019; Zhao et al., 2021) |
|  | Maternal IL-33 / ST2 signaling contributes important regulates uterine tissue remodeling and immune cell function in early pregnant mice. | (Valero-Pacheco et al., 2022) |
| Preterm labor | The IL-33/ST2 axis can induce preterm birth in pregnant mice. | (Lei et al., 2023) |
|  | ST2 is reduced on villus decidual B cells in patients with spontaneous preterm delivery. | (Huang et al., 2017) |
|  | sST2 levels in amniotic fluid were reduced in women with preterm birth. | (Stampalija et al., 2014) |
|  | The IL-33 / ST2 axis was activated in preterm women with chorioamnionitis, and the serum IL-33, ST2 levels were significantly higher than in preterm pregnant women and healthy pregnant women without chorioamnionitis. | (Cekmez et al., 2013; Lei et al., 2023) |
| Uterine leiomyoma | Serum level of IL-33 was significantly increased in patients with uterine leiomyoma. | (Santulli et al., 2013) |
|  | Serum IL-33 level was positively correlated with the total number, mass and size of myomas. | (Santulli et al., 2013) |
| Adenomyosis | Patients with adenomyosis have lower serum IL-33 levels than those in healthy women. | (Bourdon et al., 2019) |
|  | The reduced serum IL-33 level impaired the endometrial receptivity. | (He et al., 2022) |
| HPV | IL-33 expression was found in the cervix and precancerous tissues of HPV-infected women, and its expression level was negatively correlated with disease severity. | (Wang et al., 2014) |
| HSV-2 | HSV-2 infection leads to a significant increase in the expression of IL-33 in vaginal epithelial cells. | (Jin et al., 2018) |
|  | Inhibition of IL-33 release can reduce the pathological changes caused by HSV-2. | (Aoki et al., 2013) |
| HIV | Plasma levels of IL-33 and sST 2 were increased in early HIV patients | (Wu et al., 2018) |

**Reference**

Aoki, R., Kawamura, T., Goshima, F., Ogawa, Y., Nakae, S., Nakao, A., et al. (2013). Mast cells play a key role in host defense against herpes simplex virus infection through TNF-α and IL-6 production. *J Invest Dermatol* 133, 2170–2179. doi: [10.1038/jid.2013.150](https://doi.org/10.1038/jid.2013.150)

Bourdon, M., Santulli, P., Chouzenoux, S., Maignien, C., Bailly, K., Andrieu, M., et al. (2019). The Disease Phenotype of Adenomyosis-Affected Women Correlates With Specific Serum Cytokine Profiles. *Reprod Sci* 26, 198–206. doi: [10.1177/1933719118816852](https://doi.org/10.1177/1933719118816852)

Cekmez, Y., Cekmez, F., Ozkaya, E., Pirgon, O., Yılmaz, Z., Yılmaz, E. A., et al. (2013). uPAR, IL-33, and ST2 values as a predictor of subclinical chorioamnionitis in preterm premature rupture of membranes. *J Interferon Cytokine Res* 33, 778–782. doi: [10.1089/jir.2012.0151](https://doi.org/10.1089/jir.2012.0151)

He, B., Teng, X.-M., Hao, F., Zhao, M., Chen, Z.-Q., Li, K.-M., et al. (2022). Decreased intracellular IL-33 impairs endometrial receptivity in women with adenomyosis. *Front Endocrinol (Lausanne)* 13, 928024. doi: [10.3389/fendo.2022.928024](https://doi.org/10.3389/fendo.2022.928024)

Huang, B., Faucette, A. N., Pawlitz, M. D., Pei, B., Goyert, J. W., Zhou, J. Z., et al. (2017). Interleukin-33-induced expression of PIBF1 by decidual B cells protects against preterm labor. *Nat Med* 23, 128–135. doi: [10.1038/nm.4244](https://doi.org/10.1038/nm.4244)

Jin, M., Komine, M., Tsuda, H., Oshio, T., and Ohtsuki, M. (2018). Interleukin-33 is expressed in the lesional epidermis in herpes virus infection but not in verruca vulgaris. *J Dermatol* 45, 855–857. doi: [10.1111/1346-8138.14334](https://doi.org/10.1111/1346-8138.14334)

Lei, W.-J., Zhang, F., Lin, Y.-K., Li, M.-D., Pan, F., Sun, K., et al. (2023). IL-33/ST2 axis of human amnion fibroblasts participates in inflammatory reactions at parturition. *Mol Med* 29, 88. doi: [10.1186/s10020-023-00668-9](https://doi.org/10.1186/s10020-023-00668-9)

Ozler, S., Oztas, E., Guler, B. G., and Caglar, A. T. (2021). Increased levels of serum IL-33 is associated with adverse maternal outcomes in placenta previa accreta. *J Matern Fetal Neonatal Med* 34, 3192–3199. doi: [10.1080/14767058.2019.1679766](https://doi.org/10.1080/14767058.2019.1679766)

Santulli, P., Even, M., Chouzenoux, S., Millischer, A.-E., Borghese, B., de Ziegler, D., et al. (2013). Profibrotic interleukin-33 is correlated with uterine leiomyoma tumour burden. *Hum Reprod* 28, 2126–2133. doi: [10.1093/humrep/det238](https://doi.org/10.1093/humrep/det238)

Soheilyfar, S., Nikyar, T., Fathi Maroufi, N., Mohebi Chamkhorami, F., Amini, Z., Ahmadi, M., et al. (2019). Association of IL-10, IL-18, and IL-33 genetic polymorphisms with recurrent pregnancy loss risk in Iranian women. *Gynecol Endocrinol* 35, 342–345. doi: [10.1080/09513590.2018.1528220](https://doi.org/10.1080/09513590.2018.1528220)

Stampalija, T., Chaiworapongsa, T., Romero, R., Tarca, A. L., Bhatti, G., Chiang, P. J., et al. (2014). Soluble ST2, a modulator of the inflammatory response, in preterm and term labor. *J Matern Fetal Neonatal Med* 27, 111–121. doi: [10.3109/14767058.2013.806894](https://doi.org/10.3109/14767058.2013.806894)

Valero-Pacheco, N., Tang, E. K., Massri, N., Loia, R., Chemerinski, A., Wu, T., et al. (2022). Maternal IL-33 critically regulates tissue remodeling and type 2 immune responses in the uterus during early pregnancy in mice. *Proc Natl Acad Sci U S A* 119, e2123267119. doi: [10.1073/pnas.2123267119](https://doi.org/10.1073/pnas.2123267119)

Wang, L., Li, H., Liang, F., Hong, Y., Jiang, S., and Xiao, L. (2014). Examining IL-33 expression in the cervix of HPV-infected patients: a preliminary study comparing IL-33 levels in different stages of disease and analyzing its potential association with IFN-γ. *Med Oncol* 31, 143. doi: [10.1007/s12032-014-0143-0](https://doi.org/10.1007/s12032-014-0143-0)

Wu, X., Li, Y., Song, C.-B., Chen, Y.-L., Fu, Y.-J., Jiang, Y.-J., et al. (2018). Increased Expression of sST2 in Early HIV Infected Patients Attenuated the IL-33 Induced T Cell Responses. *Front Immunol* 9, 2850. doi: [10.3389/fimmu.2018.02850](https://doi.org/10.3389/fimmu.2018.02850)

Yue, J., Tong, Y., Xie, L., Ma, T., and Yang, J. (2016). Genetic variant in IL-33 is associated with idiopathic recurrent miscarriage in Chinese Han population. *Sci Rep* 6, 23806. doi: [10.1038/srep23806](https://doi.org/10.1038/srep23806)

Zhao, L., Fu, J., Ding, F., Liu, J., Li, L., Song, Q., et al. (2021). IL-33 and Soluble ST2 Are Associated With Recurrent Spontaneous Abortion in Early Pregnancy. *Front Physiol* 12, 789829. doi: [10.3389/fphys.2021.789829](https://doi.org/10.3389/fphys.2021.789829)

Zidan, H. E., Abdul-Maksoud, R. S., Mowafy, H. E., and Elsayed, W. S. H. (2018). The association of IL-33 and Foxp3 gene polymorphisms with recurrent pregnancy loss in Egyptian women. *Cytokine* 108, 115–119. doi: [10.1016/j.cyto.2018.03.025](https://doi.org/10.1016/j.cyto.2018.03.025)
